# Coronary microvascular dysfunction in heart failure patients

**DOI:** 10.3389/fcvm.2023.1153994

**Published:** 2023-06-02

**Authors:** Takumi Toya, Yuji Nagatomo, Yukinori Ikegami, Nobuyuki Masaki, Takeshi Adachi

**Affiliations:** Division of Cardiology, National Defense Medical College, Tokorozawa, Saitama, Japan

**Keywords:** coronary physiology, coronary microvascular dysfunction, heart failure with reduced ejection fraction, heart failure with preserved ejection fraction, coronary flow reserve

## Abstract

Coronary microcirculation has multiple layers of autoregulatory function to maintain resting flow and augment hyperemic flow in response to myocardial demands. Functional or structural alterations in the coronary microvascular function are frequently observed in patients with heart failure with preserved or reduced ejection fraction, which may lead to myocardial ischemic injury and resultant worsening of clinical outcomes. In this review, we describe our current understanding of coronary microvascular dysfunction in the pathogenesis of heart failure with preserved and reduced ejection fraction.

## Introduction

**Figure 1:**
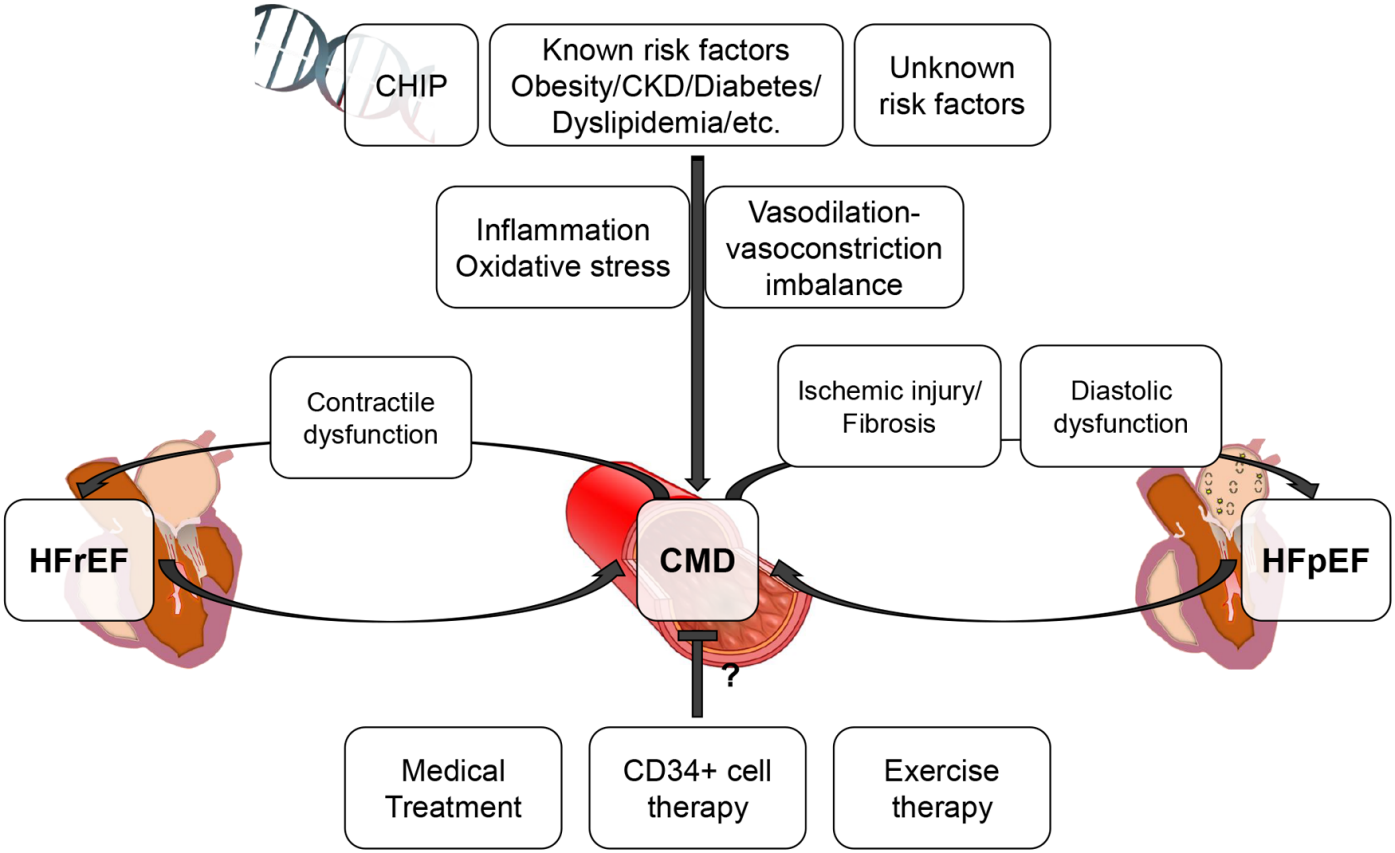
Conceptual association between CMD and HFpEF/HFrEF. This figure illustrates the conceptual association between CMD and HFpEF/HFrEF. CMD can be involved in the progression of HFpEF/HFrEF and associated with worse prognosis. CHIP, clonal hematopoiesis of indeterminate potential; CKD, chronic kidney disease; CMD, coronary microvascular dysfunction; HFpEF, heart failure with preserved ejection fraction; HFrEF, heart failure with reduced ejection fraction.

Percutaneous revascularization for epicardial coronary artery disease (CAD) as an initial treatment strategy did not improve survival even in chronic coronary syndrome patients with evidence of moderate-severe ischemia (ISCHEMIA trial) (1). Revascularizing epicardial CAD with percutaneous coronary intervention (PCI) also failed to show additional benefit in reducing all-cause death or heart failure hospitalization beyond optimal medical therapy in patients with left ventricular systolic dysfunction (REVIVED-BCIS2) (2). Given the fact that the myocardial oxygen extraction from the coronary circulation is approximately 70%–80% at rest and that increased myocardial oxygen demands need to be met by increasing coronary flow (3, 4), the above mentioned negative results of epicardial revascularization may potentially underline the importance of the coronary microvasculature to augment coronary flow to meet the myocardial demands. Recently, coronary microvascular dysfunction has been rigorously studied and appears to be involved not only in the development of ischemic symptoms but also in various pathological processes and conditions, including heart failure (5–8). In this short review, we will discuss the relationship between coronary microvascular dysfunction and heart failure with preserved or reduced ejection fraction (Figure 1).

## Incidence of CMD in patients with HFpEF

With recent advances in imaging modalities and techniques, it has been uncovered that coronary microvascular dysfunction (CMD) can frequently manifests in patients with heart failure with preserved ejection fraction (HFpEF). PROMIS-HFpEF (Prevalence of Microvascular Dysfunction in HFpEF) trial is a prospective multinational multi-center observational study enrolling 202 patients with HFpEF (left ventricular ejection fraction [LVEF] ≥40%), revealing that 75% of patients with HFpEF had CMD defined as adenosine stress transthoracic Doppler echocardiography-derived coronary flow reserve (CFR) <2.5 in the absence of unrevascularized epicardial CAD (9). One-year outcomes in patients from PROMIS-HFpEF trial showed that HFpEF patients with CMD had a significantly higher incidence rates of cardiovascular death and heart failure hospitalization than those without. Even though adverse events were detected in only 15 patients within 1 year, CMD was an independent risk factor for cardiovascular death and heart failure hospitalization (10). A caveat of this study was that the CFR measurements were performed non-invasively with echocardiography. However, another group performed invasive assessments of CMD to separately evaluate endothelium-dependent and endothelium-independent CMD, and reported a consistent prevalence of CMD in patients with HFpEF. Among 162 HFpEF patients (LVEF ≥50%), 29% had isolated endothelium-dependent CMD (defined as an increase of coronary blood flow ≤0% in response to incremental doses of intracoronary acetylcholine [10^−6^, 10^−5^, and 10^−4 ^mol/L]: %change in CBF), 33% had isolated endothelium-independent CMD (defined as CFR <2.5 in response to incremental doses of intracoronary adenosine [18–72 μg]), and 10% had combined CMD, leaving a total of 72% with abnormal coronary microvascular function. Importantly, no single clinical characteristics could identify the presence of either endothelium-dependent or endothelium-independent CMD. Both endothelium-dependent and endothelium-independent CMD were associated with higher all-cause mortality; however, patients with endothelium-dependent CMD displayed a greater left ventricular end-diastolic dimension, while those with endothelium-independent CMD displayed impaired left ventricular diastolic function with a higher E/e’ ratio, indicating that both pathways have different effects on the pathophysiology of HFpEF (11).

## Effects of CMD on hemodynamics during exercise and on myocardial injury in HFpEF

The effects of the endothelium-dependent and endothelium-independent CMD on hemodynamics during exercise were rigorously analyzed in 51 patients who underwent exercise right heart catheterization and concurrent invasive coronary reactivity testing for assessment of unexplained exertional symptoms in the absence of obstructive CAD (<50% stenosis) and impaired systolic left ventricular function (LVEF >50%). Pulmonary artery wedge pressure (PAWP) at peak exercise was significantly higher in patients with endothelium-dependent CMD or endothelium-independent CMD than those without, with a significant negative correlation between mean PAWP at peak exercise and %change in CBF in response to acetylcholine (an indicator of endothelium-dependent coronary microvascular function) or CFR (an indicator of endothelium-independent coronary microvascular function). Furthermore, this study uniquely demonstrated that higher CFR was significantly correlated with a greater exercise capacity, while there was no significant correlation between %change in CBF and exercise capacity, underlining the mechanistic importance of endothelium-independent pathway in HFpEF pathology (12). In fact, CFR <2.0 assessed with positron emission tomography was significantly associated with E/e′>15, which is an echocardiographic feature of left ventricular diastolic dysfunction in 201 patients with normal LVEF in the absence of significant epicardial CAD. Negative correlation between CFR and E/e′ was more pronounced in patients with troponin elevation compared to those without. Moreover, patients with both CFR <2 and E/e′ >15 had significantly higher incidence of HFpEF hospitalization than the other groups, indicating a causal link between myocardial ischemic injury due to CMD and diastolic dysfunction, the main underlying pathology of HFpEF (13). Another study assessing myocardial oxygen supply and demand calculated from invasively obtained PAWP and aortic pressure waveform at rest and during exercise demonstrated that HFpEF patients had markedly decreased myocardial oxygen supply which augmented during exercise, leading to an elevated oxygen supply-demand mismatch. The greater the oxygen supply-demand mismatch, the higher the troponin levels during exercise, suggesting a vicious cycle in which ischemic myocardial damage induces diastolic dysfunction, which in turn induces further myocardial damage through oxygen supply-demand mismatch (14). In contrast to previous studies, which have reported the link between myocardial ischemic injury induced by CMD and the development of HFpEF, Arnold et al. demonstrated that myocardial perfusion reserve, as assessed by cardiac magnetic resonance imaging, was not significantly associated with either late gadolinium enhancement or extracellular volume - markers of myocardial fibrosis (15). These inconsistent findings may be due to the fact that both CMD and HFpEF are heterogeneous conditions for which a single underlying mechanism cannot fully account.

## Vascular dysfunction as a potential link between atrial fibrillation and HFpEF

Atrial fibrillation (AF) is frequently observed in patients with HFpEF, and its presence has been associated with poorer long-term clinical outcomes (16, 17). Comorbid AF is more strongly correlated with incident HFpEF than with heart failure with reduced ejection fraction (HFrEF) (18). Additionally, studies have demonstrated that left atrial compliance and function progressively deteriorate with increasing AF burden in HFpEF patients during the transition from paroxysmal to permanent AF (19). These findings lend support to the hypothesis that AF may serve as an indicator of the underlying pathophysiological processes in patients with HFpEF. One potential mechanism linking AF and HFpEF is endothelial dysfunction (20). A previous study demonstrated that patients with epicardial and/or coronary microvascular endothelial dysfunction had a 5.8-fold increased relative risk of developing AF compared to those without coronary endothelial dysfunction (21). Indeed, inflammatory endothelial activation characterized by upregulation of E-selectin and intercellular adhesion molecule was observed in HFpEF patients, accompanied by increased oxidative stress in the endothelial cells and impaired NO-dependent signaling in the myocardium, potentially leading to cardiomyocyte stiffening and hypertrophy in HFpEF patients (22, 23). Inflammation in the heart or systemic circulation may in turn alter the electrophysiology and structural substrate of the atria, thus increasing their vulnerability to atrial fibrillation (24). The potential interplay between vascular dysfunction and AF development in HFpEF warrants further investigation.

## Risk factors and potential treatment of CMD in HFpEF patients

Endothelium-independent CMD, characterized by a decline in coronary flow reserve - defined as the ratio of hyperemic flow to resting flow - can be attributed to either increased resting flow or decreased hyperemic flow. The former subtype has been linked to dysfunctional autoregulation of the coronary microvasculature, while the latter has been associated with structural microvascular alterations in CMD. In diabetic patients, a decline in CFR has been observed in conjunction with increasing resting coronary flow (25, 26). In a swine model of CMD induced by multiple risk factors (diabetes mellitus, hypercholesterolemia, and chronic kidney disease), a decline in CFR due to increased resting flow was observed, and myocardial efficiency was found to be lower, requiring higher oxygen consumption for a given level of myocardial work (27). This animal model also exhibited left ventricular diastolic dysfunction, a key feature of HFpEF, potentially through systemic inflammation and oxidative stress accompanied by metabolic alterations in glucose and fatty acid (28, 29). Interestingly, a recent study reported a possible beneficial effect of sodium-glucose cotransporter 2 inhibitors on CMD by decreasing resting coronary flow and thereby increasing myocardial flow reserve in diabetic patients (30). However, another study assessing the effects of sodium-glucose cotransporter 2 inhibitors on microvascular flow reserve in diabetic patients failed to improve it (31). The difference between these two studies is that the former included diabetic patients with diabetes duration of less than 10 years, while the latter included those with a median diabetes duration of more than 10 years. One study indicated that CMD subtypes could alter from functional to structural as the duration of diabetes exceeds ten years (32). The underlying pathophysiology of the decrease in CFR may differ between patients with increased resting flow (functional CMD) and those with decreased hyperemic flow (structural CMD), both of which equally contribute to worsening clinical outcomes (33); however, response to therapeutic agents can vary between these two distinct CMD subtypes. Obesity has also been linked to functional and structural CMD in clinical and experimental studies (34). Vasodilation-vasoconstriction imbalance associated with obesity can lead to functional microvascular vasomotor dysfunction (35). Further, histological analyses showed that coronary capillary rarefaction was observed in obese patients and animals (36–38). Chronic kidney disease is another risk factor that has induced CMD and HFpEF in experimental animals. A large community-based cohort study has also shown an association between chronic kidney disease and new-onset HFpEF (39). Impaired renal clearance of uremic toxins can lead to a chronic inflammatory state and systemic endothelial dysfunction, including coronary microvascular endothelial dysfunction (40). Coronary endothelial cells can modulate left ventricular diastolic function via the paracrine effect (41); therefore, coronary microvascular endothelial dysfunction with parallel pathological changes in systemic endothelium can be the link between chronic kidney disease and HFpEF. Various studies are ongoing to evaluate drug therapy for CMD. The CorMicA trial reported the efficacy of stratified medical treatment based on the assessment using invasive coronary function testing in patients with angina and no obstructive CAD (42, 43). However, there is currently no established treatment selection based on different CMD subtypes. Furthermore, data is lacking on whether CMD can be a therapeutic target for HFpEF patients (44). In fact, rarefaction of the coronary microvasculature has been observed in postmortem HFpEF patients, and the extent of coronary rarefaction has been found to correlate with myocardial fibrosis (45). CMD patients with advanced structural alterations may have limited benefits from pharmacotherapy. Recently published phase II trials reported the beneficial effects of CD34 + cell therapy on CMD, suggesting that CD34 + cell therapy may be a promising new treatment strategy for CMD patients with advanced structural microvascular alterations potentially through angiogenesis (46–48). In addition to CMD subtypes, sex differences should also be considered. Women with angina and no obstructive CAD were shown to have lower CFR due to higher resting coronary flow compared to men, implicating the sex difference in coronary physiology (49). A recent study that performed sex-specific proteomic profiling associated with CMD in patients with HFpEF from PROMIS-HFpEF trial demonstrated that CMD was related to the inflammation-mediated chemokine and cytokine signaling pathway among men with HFpEF, and the P13-kinase and transforming growth factor-beta signaling pathway among women with HFpEF. Thus, reduction of inflammation can potentially be more important than neurohormonal modulation in men, while neurohormonal blockade may be more effective in women to prevent left ventricular remodeling and myocardial fibrosis (50). The previous observation that treatment with sacubitril-valsartan more significantly reduces heart failure hospitalizations in women than in men with HFpEF supports this hypothesis (51). A recent finding related to inflammation is the interesting association between clonal hematopoiesis of indeterminate potential (CHIP), particularly in *TET2* and *DNMT3A*, and an increased risk of cardiovascular disease, possibly via the interleukin-6 (IL-6) signaling pathway (52, 53). Another study evaluating the association between CMD and CHIP reported that CHIP in *TET2* and *DNMT3A* is an independent risk factor for CMD, with CHIP mediating 32% of the increased risk of major adverse cardiovascular events in CMD patients (54). Higher levels of IL-6 were associated with all-cause or cardiovascular death and heart failure hospitalization in patients recently hospitalized with HFpEF, even after adjustment for other risk factors (55). These data may highlight anti-inflammatory therapy as a potential therapeutic target for HFpEF and CMD. Finally, as a non-pharmacologic therapy, there is growing evidence regarding exercise therapy for patients with HFpEF (56). Although exercise training is associated with improved peak oxygen uptake and exercise tolerance in patients with HFpEF, a meta-analysis of randomized controlled trials showed that improvements in cardiorespiratory function and quality of life with exercise training were not accompanied by improvements in left ventricular systolic and diastolic dysfunction in HFpEF patients (57). A recent study examining the effects of exercise therapy showed no change in peripheral vascular function or endothelial repair capacity despite improvement in peak oxygen uptake with aerobic exercise training (58). Further studies are needed to determine the effects of exercise training on coronary microvascular function.

## CMD and HFrEF

There is scarce evidence for an association between CMD and HFrEF in comparison to HFpEF. Given the systemic nature of microvascular disease (59–64), the fact that diabetic microvascular complications (neuropathy, nephropathy, and retinopathy) were associated with worse clinical outcomes in patients with HFpEF or HFrEF may indicate the pathophysiologic roles of CMD on both HFpEF and HFrEF (65). Notably, the accumulation of microvascular complications has been associated with an increased incidence of cardiac hypertrophy in patients with HFpEF, while it has been associated with a decreased incidence of cardiac hypertrophy in patients with HFrEF, indicating the differential impact of CMD on HFpEF and HFrEF (65). In fact, a previous study assessing the correlation between regional coronary flow reserve (CFR) and contractile reserve in patients with dilated cardiomyopathy revealed a significant positive correlation between CFR and contractile reserve in the left anterior descending and left circumflex territories. Furthermore, decreased CFR was correlated with increased left ventricular end-diastolic pressure in the same regions, highlighting the importance of microvascular reserve in maintaining myocardial contractile function (66). Small studies have evaluated the impact of CFR on clinical outcomes in patients with HFrEF and have consistently shown that abnormal CFR predicts worse clinical outcomes (67, 68). A retrospective study including 510 HFrEF patients who underwent rest/stress myocardial perfusion positron emission tomography to quantify CFR found that lower CFR (≤1.65) was associated with a two-fold increased risk of major adverse cardiovascular events compared to those with higher CFR (>1.65), supporting previous observations in a larger cohort (69). When factors contributing to decreased CFR are divided into structural CMD due to decreased hyperemic flow and functional CMD due to increased resting flow, it has been reported that decreased hyperemic flow increases the relative risk of death and heart failure onset and progression by 3.5 times (70). However, a recent report from a large registry data demonstrated that structural and functional CMD contributed equally to worsening clinical outcomes. Notably, CFR was lower in patients with reduced LVEF than those with preserved LVEF, primarily due to increased resting flow. The mechanism of increased resting flow in patients with reduced LVEF is unclear; however, it may be attributed to increased left ventricular end-diastolic pressure and increased myocardial oxygen demand due to elevated heart rate and left ventricular mass (71).

## Summary and future perspectives

Coronary microvascular dysfunction can often coexist in heart failure patients with preserved and reduced ejection fractions. Although the underlying pathophysiology of functional or structural CMD may differ, both may equally contribute to worse clinical outcomes. There is a paucity of available data on whether comorbid CMD is a potential target for heart failure therapy. Furthermore, it needs to be clarified whether different CMD subtypes respond differently to a treatment. However, further phenotypic assessment of heart failure patients may pave the way to a clearer understanding of the pathogenesis of heart failure and the unmet clinical needs of heart failure patients.
